# Sirtuin 1 is a key molecular link between cellular senescence and heart failure

**DOI:** 10.3389/fmmed.2026.1818104

**Published:** 2026-04-28

**Authors:** Jan Krekora, Jarosław Drożdż, Janusz Blasiak

**Affiliations:** 1 2nd Department of Cardiology, Medical University of Lodz, Lodz, Poland; 2 Faculty of Medicine, Collegium Medicum, Mazovian Academy in Plock, Plock, Poland

**Keywords:** cellular senescence, DNA damage, heart failure, mitochondrial impairment, myocardial hypertrophy, oxidative stress, senescence-associated secretory phenotype, sirtuin 1

## Abstract

Cellular senescence, a state where cells permanently exit the cycle but remain metabolically active, plays a role in cardiovascular diseases (CVD), including heart failure (HF). Senescent cells accumulate in aging and stressed heart tissue, releasing pro-inflammatory cytokines, chemokines, and enzymes that affect endothelial cells. This ongoing inflammation exacerbates cardiac damage, induces endothelial dysfunction, and triggers secondary senescence across various cardiac cell types. Senescent cardiomyocytes contribute to reduced systolic function by causing mitochondrial damage, impaired contractility, and metabolic dysfunction, leading to lower cardiac output and symptoms such as fatigue and exercise intolerance in HF patients. Additionally, inflammation from senescent endothelial cells and loss of microvasculature impair coronary blood flow and oxygen delivery, worsening symptoms such as shortness of breath, angina-like discomfort, and fluid retention by disrupting cardiac energy metabolism and increasing filling pressures. A key factor linking cellular senescence with HF is sirtuin 1 (SIRT1), a histone deacetylase with antioxidant activity. SIRT1 acts as a hormetic regulator in the heart, being beneficial within a narrow range but potentially harmful when overstimulated. As drugs targeting senescence are emerging to treat CVD, it is important to evaluate how SIRT1 may influence the connection between senescence and HF to improve anti-senescent therapies. In this narrative/perspective review, we explore the molecular mechanisms underlying senescence in HF development and how SIRT1 might modulate these processes for therapeutic benefit.

## 1 Introduction

Heart failure (HF) poses a significant global public health challenge, causing serious economic, social, and clinical impacts. It is estimated that the worldwide economic burden of HF reached $284.17 billion in 2021, with nearly equal shares from direct medical expenses (48.16%) and indirect costs, such as lost productivity (51.84%) (Darvish et al., 2025). In clinical practice, HF remains a leading cause of hospitalization, with up to 80% of HF patients experiencing repeated admissions (Inam et al., 2025). Beyond economic costs, HF affects patients’ social and functional wellbeing and has an estimated 50% five-year mortality rate, emphasizing its progressive and life-limiting nature (Johansson et al., 2021).

Diagnostically, HF is highly complex due to its diverse clinical phenotypes, including reduced, preserved, and mid-range ejection fraction (HFrEF, HFpEF, and HFmrEF), as well as various comorbidity clusters, such as frailty and polypharmacy profiles. Symptoms such as dyspnea, edema, and chronic fatigue are nonspecific and can overlap with respiratory, renal, and metabolic conditions, making early detection more difficult. The high prevalence of comorbidities further delays accurate diagnosis (McDonagh et al., 2021).

Despite advances in guideline-directed medical therapy (GDMT) and the availability of evidence-based treatments, HF therapy still faces significant challenges, and its implementation is often suboptimal due to polypharmacy, frailty, and limited access to specialist care (Ambardekar and Rafei, 2023). Central to improving outcomes are multidisciplinary models like Heart Failure Disease Management Programs (HF-DMPs), which combine medication optimization, telemonitoring, transitional care, and social support (AlHabeeb, 2022).

One reason that current therapeutic approaches in HF remain insufficient for many patient subgroups may be poor knowledge of the molecular mechanisms underlying this disease. Heart failure is a complex syndrome caused by various cellular and molecular abnormalities (Fonseka et al., 2025). While progress in neurohormonal blockade has improved outcomes, particularly in HFrEF, advancements in HFpEF have been limited due to its diverse, incompletely understood mechanisms (Yang Z. et al., 2025). This variability underscores the need to understand the molecular pathways underlying HF to develop targeted, phenotype-specific treatments.

Several key biological processes contribute to HF development and progression, including neurohormonal activation, metabolic remodeling, mitochondrial dysfunction, inflammation, fibrosis, calcium dysregulation, and microvascular impairment (Zhang F. et al., 2025). However, how these processes interact precisely remains only partially understood. Therefore, detailed molecular studies are essential for clarifying these mechanisms and discovering new therapeutic targets. Importantly, HF is the final pathway for various cardiovascular disorders, such as dilated and hypertrophic cardiomyopathy, hypertension, arrhythmias, and myocardial infarction (MI), each involving different signaling networks (Zeng et al., 2025). Understanding how these upstream conditions lead to common heart failure phenotypes requires integrated molecular approaches. Additionally, the limited success of current treatments and the lack of therapies that reduce mortality in HFpEF reveal a gap between clinical syndromes and their molecular foundations (Kondo et al., 2024). As HF prevalence rises, driven by aging populations and increasing metabolic and inflammatory comorbidities, ongoing molecular research is vital to reducing global morbidity and mortality. The interactions among structural remodeling, neurohormonal dysregulation, oxidative stress, and immune activation are complex and can be fully understood only through mechanistic studies at the cellular and molecular levels.

Cellular senescence plays a key pathogenic role in the development and progression of HF (Zhao et al., 2025). Senescent cells accumulate in the aging and diseased cardiovascular system and act as an independent risk factor for major cardiovascular conditions, including hypertension, myocardial infarction, and HF (Xu et al., 2025). They exert harmful effects primarily through the senescence-associated secretory phenotype (SASP), which releases pro-inflammatory cytokines, chemokines, and proteases that promote tissue dysfunction, fibrosis, mitochondrial impairment, and oxidative stress–key effects that drive adverse cardiac remodeling (Sutanto et al., 2025). Aging-related senescent cardiomyocytes, endothelial cells, fibroblasts, and vascular smooth muscle cells contribute to the deterioration of myocardial structure and function, thereby creating a microenvironment that promotes HF progression (Wu et al., 2025). Senescent cells not only accumulate due to impaired clearance in aging tissues but also exacerbate disease progression by disrupting repair processes and promoting metabolic and epigenetic changes that create a vicious cycle of cardiovascular decline. As a result, strategies targeting senescence, such as senolytics and senomorphics, have gained significant research interest as potential treatments for HF. However, senolytic therapy for cardiovascular diseases remains experimental.

Sirtuin 1 (SIRT1), a class III nicotinamide adenine dinucleotide (NAD^+^)–dependent histone deacetylase, is a central regulator of cardiac aging and plays a protective role in HF pathogenesis by maintaining mitochondrial homeostasis, reducing oxidative stress, and modulating metabolic and inflammatory signaling (Chen S et al., 2025; Cozzolino et al., 2025). Dysregulation of mitochondrial function is a major driver of cardiac aging and HF, and SIRT1 helps preserve mitochondrial quality control, energy production efficiency, and antioxidant defenses (Sancak et al., 2025). Reduced SIRT1 activity, often associated with aging, metabolic disturbances, and declining NAD^+^ levels, leads to impaired mitochondrial function, increased oxidative injury, and enhanced susceptibility to HF-related structural remodeling (Wei et al., 2025). By regulating processes such as DNA repair, stress resistance, and inflammatory pathways, SIRT1 helps counteract key cellular mechanisms that contribute to HF progression (Ding et al., 2025). Moreover, pharmacological or nutritional activation of SIRT1 has shown promise in attenuating cardiac aging and HF-related dysfunction by improving mitochondrial performance and reducing pathological signaling, making it an attractive target for future HF therapies (Zhang et al., 2024).

SIRT1 suppresses cellular senescence by deacetylating key regulators of aging and stress responses, thereby preserving mitochondrial function and reducing inflammatory signaling that accelerates HF progression (Sung et al., 2021). Conversely, increased senescence reduces SIRT1 activity, creating a vicious cycle that promotes cardiomyocyte dysfunction and adverse remodeling in HF (Hayakawa et al., 2015). Therefore, understanding the interplay between SIRT1 and cellular senescence may be important for HF therapy, as it could inform strategies that target this interplay to treat or prevent HF.

In this critical review, we present information on SIRT1, cellular senescence and their role in cardiovascular disease (CVD) pathogenesis. Further, we focus on the mutual interaction between SIRT1 and cellular senescence, its role in HF pathogenesis, and perspectives for exploiting this interaction in HF treatment. Although several excellent reviews address the role of SIRT1 in cardiovascular diseases and explore the involvement of cellular senescence in these conditions, the role of SIRT1 in senescence-mediated pathogenesis of HF has not been addressed so far.

## 2 Sirtuin 1 and its role in heart failure

Sirtuin 1 belongs to the sirtuin family, including seven members (SIRT1–SIRT7), which are class III histone deacetylases (HDACs). It requires NAD^+^ as the essential and obligate cofactor and is encoded by the SIRT1 gene, located on chromosome 10 and spanning chr10: 67,884,656-67,918,390 Mb (GRCh38/hg38) (SIRT1, 2026). The SIRT1 gene has two major promoters: promoter 1 (distal) drives transcription from exon 1a, and promoter 2 (proximal) drives transcription from exon 1b. Exons 1a and 1b are alternative first exons. Both promoters generate mRNAs that encode the same SIRT1 protein because alternative first exons splice into the same downstream coding exons. Transcription factors that activate SIRT1 expression are forkhead box O1/O3 (FOXO1/3), cAMP response element-binding protein (CREB), peroxisome proliferator-activated receptor alpha (PPARα), peroxisome proliferator-activated receptor gamma coactivator-1 alpha (PGC-1α), and hypoxia-inducible factor 2 alpha (HIF-2α), although in some cases, this regulation is effective only in specific tissues (Yang et al., 2022). Transcription factors that repress SIRT1 expression are p53, hypermethylated in cancer 1 (HIC1), signal transducer and activator of transcription 3 (STAT3), and nuclear factor kappa-light-chain-enhancer of activated B cells (NF-κB). Epigenetic regulation of the SIRT1 promoter includes DNA methylation, H3K9/H3K27 methylation, and histone acetylation (Yang et al., 2022).

SIRT1 expression is regulated through multiple, layered mechanisms that integrate nutrient status, stress signals, hormones, and epigenetic states (Sun et al., 2024). Its regulation occurs at four major levels–transcriptional (see above), post-transcriptional (miRNAs, RNA-binding proteins), post-translational (protein stability, histone modification) and metabolic.

SIRT1 affects gene expression primarily by deacetylating histones and key transcription regulators in an NAD^+^-dependent manner (Zhang and Kraus, 2010). SIRT1 removes acetyl groups from specific histone lysines, especially H3K9ac, H3K14ac, and H4K16ac, which may lead to chromatin compaction, reduced accessibility for transcription factors, and resulting suppressed transcription. Besides these chromatin-mediated effects on gene expression, SIRT1 may deacetylate transcription factors and co-regulators, including NF-κB, p53, FOXOs, PGC-1α (Jiang et al., 2024). SIRT1 may affect gene expression in several other pathways, including cooperation with epigenetic complexes, resulting in long-term gene regulation and linking metabolism with transcription through its NAD^+^ dependence (Fan and Li, 2023; Ryall et al., 2015; Wang et al., 2019).

SIRT1 is widely expressed throughout the cardiovascular system, but its levels, cellular distribution, and functional consequences vary by cell type and physiological context (Dinislam et al., 2025). The highest expression of SIRT1 occurs in endothelial cells (ECs) (Potente and Dimmeler, 2008). This reflects the need for ECs to regulate nitric oxide production, the anti-inflammatory program, the antioxidant response, angiogenesis, and mitochondrial homeostasis. SIRT1 inhibits endothelial senescence and atherosclerosis progression (Zu et al., 2010). Reduced endothelial SIRT1 is associated with hypertension, coronary artery disease, diabetes, and endothelial dysfunction (Chan et al., 2017; Man et al., 2019; Orimo et al., 2009). SIRT1 plays a dual, context-dependent role in HF–at physiological or moderately increased levels, it is cardioprotective, while at very high levels, especially during chronic stress, SIRT1 can become maladaptive (Zhou et al., 2024). SIRT1 supports cardiomyocytes during pressure overload, ischemia, and aging by increasing mitochondrial biogenesis, oxidative phosphorylation efficiency, and fatty-acid oxidation through deacetylation and activation of PGC-1α. These effects are beneficial for HF, as they reduce mitochondrial dysfunction and the resulting reactive oxygen and nitrogen species (RONS) overproduction, and slow the progression towards systolic or diastolic dysfunction. SIRT1 may reduce cardiomyocyte apoptosis by deacetylating p53 and FOXO3a, thereby limiting cardiomyocyte loss, protecting against post-infarction remodeling, and preserving systolic function. SIRT1 inhibits cardiac hypertrophic pathways by decreasing protein kinase B (AKT)-mechanistic target of rapamycin (MTOR) signaling and nuclear factor kappa-light-chain-enhancer of activated B cells (NF-κB)-driven inflammation, and by increasing AMP-activated protein kinase (AMPK)-dependent metabolic reprogramming (Wang et al., 2023). In consequence, it attenuates concentric hypertrophy and slows progression to HFpEF. SIRT1 improves vascular function, indirectly supporting heart homeostasis by reducing myocardial stress and ventricular remodeling via improved coronary perfusion, reduced systemic blood pressure, and lower cardiac afterload.

Aging, diabetes, obesity, and chronic inflammation decrease SIRT1 expression/activity, causing several HF-related effects (Ministrini et al., 2021; Ruperez et al., 2025). Reduced SIRT1 activity decreases PGC-1α signaling, increases RONS production, and causes mtDNA damage, driving HF in both pressure overload and metabolic cardiomyopathy. Low SIRT1 levels enhance transforming growth factor (TGF-β)/SMAD signaling in cardiac fibroblasts, leading to excessive EM deposition and stiff ventricles–effects especially relevant in HFpEF. Reduced SIRT1 activity leads to increased NF-κB acetylation and activation, resulting in cytokine release, endothelial dysfunction, and adverse remodeling. SIRT1 is a major activator of autophagy (via ATG proteins and FOXO), and low SIRT1 causes impaired clearance of damaged mitochondria that may result in cardiomyocyte death (Hariharan et al., 2010).

A decrease in SIRT1 expression or activity can contribute to cardiovascular pathology, whereas excessively high SIRT1 overexpression and activity may also have harmful effects. In a mouse model of HF, SIRT1 levels were 5-7-fold higher than normal, leading to mitochondrial depletion, abnormal fatty acid metabolism, dilated cardiomyopathy, and increased oxidative stress (Alcendor et al., 2007; Kawashima et al., 2010). The mechanisms underlying these effects may include disturbances in calcium-handling proteins and overactivation of PGC-1α, leading to mitochondrial overexpansion and metabolic imbalance. Excessive autophagy may lead to the loss of contractile proteins. Therefore, too little or too much SIRT may contribute to HF pathogenesis, whereas optimal SIRT1 may protect against this syndrome.

Low SIRT1 levels contribute to myocardial stiffness, endothelial dysfunction, perivascular inflammation, and hypertensive remodeling in HFpEF (Li et al., 2019). SIRT1 may mediate improvements in these pathways through caloric restriction, exercise, and NAD + boosters, including nicotinamide riboside (NR) and nicotinamide mononucleotide (NMN) (Lagunas-Rangel, 2025).

Because the SIRT1-inducing effects depend specifically, non-monotonically, on its activity, it is important to provide more details on this issue from the perspective of using SIRT1-modulating agents in HF therapy. Moderate SIRT1 co-activates PGC-1α, enhancing mitochondrial biogenesis and oxidative capacity while maintaining balance with antioxidant defense via FOXO, supporting high energy demand in pressure overload or aging without excessive RONS (Alcendor et al., 2007). Excess SIRT1, however, disrupts metabolic programs: in hearts with overexpressed SIRT1, fatty-acid uptake and mitochondrial respiration decrease, and degenerated mitochondria accumulate, indicating that global deacetylation shifts metabolism away from efficient oxidative fuel use and healthy organelle turnover (Kawashima et al., 2010). With low (2.5-fold) to moderate (7.5-fold) overexpression of SIRT1, FOXO-dependent induction of antioxidant enzymes, including catalase, enhances stress resistance and reduces net RONS, which maintains contractile function (Alcendor et al., 2007). With very high SIRT1 levels (12.5-fold), basal oxidative stress increases, and apoptosis and hypertrophy rise, leading to cardiomyopathy. Physiological upregulation of SIRT1, such as under mild stress, fine-tunes deacetylation of a limited set of substrates (PGC-1α, FOXOs, p53), yielding adaptive hypertrophy resistance and preserved function. Supraphysiologic SIRT expression broadens deacetylation to many targets, represses FA metabolism programs, and impairs mitochondrial respiration–changes that are detrimental at the organ level (Kawashima et al., 2010). Therefore, SIRT1 behaves as a hormetic regulator in the heart–beneficial within a tight window and harmful when over-activated. Mouse genetics show clear thresholds: protection up to ∼7.5-fold; cardiomyopathy around ≥12.5-fold.

Higher SIRT1 activity was found in peripheral blood mononuclear cells (PBMCs) isolated from patients with HFrEF compared to those with mid-range ejection fraction (HFmrEF), and even more so compared to those with HFpEF (Conti et al., 2020). Because SIRT1 levels were found to differ sufficiently to distinguish patients with HFmrEF/HFrEF from those with HFpEF, it has been hypothesized that SIRT1 may also serve as a useful biomarker for selecting the best therapy based on HF phenotype.

## 3 Cellular senescence and cardiovascular diseases

Cellular senescence, further referred to as senescence, unless otherwise stated, is a state in which a cell is permanently withdrawn from the cell cycle and stops dividing despite retaining metabolic activity. Many factors may induce senescence, including telomere erosion (replicative senescence) due to the end-replication problem (Victorelli and Passos, 2017).

**FIGURE 1:**
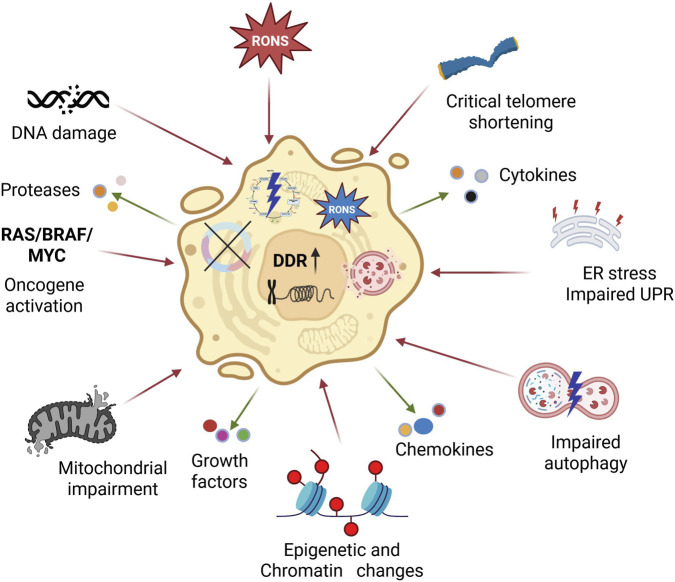
Cellular senescence can be induced by various stresses, including oxidative stress associated with overproduction of reactive oxygen and nitrogen species (RONS), telomere erosion, endoplasmic reticulum (ER) stress associated with impaired unfolded protein response (UPR), oncogene activation, here represented by rat sarcoma viral oncogene homolog (RAS), v-raf murine sarcoma viral oncogene homolog B1 (BRAF), and MYC proto-oncogene, bHLH transcription factor (MYC), mitochondrial impairment, autophagy decline, changes in chromatin structure with alterations of the epigenetic profile. Senescent cells are permanently withdrawn from the cell cycle and display a senescence-associated secretory phenotype (SASP), characterized by the release of signaling molecules, including RONS, cytokines, chemokines, proteases, and growth factors, either directly or via extracellular vehicles, that induce permanent inflammation and tissue remodeling. DNA damage is a prototype of cellular senescence inducers, and senescent cells are characterized by a permanently ongoing DNA damage response (DDR). Created in https://BioRender.com.

Other factors to induce senescence are stress, including oxidative stress associated with overproduction of RONS and endoplasmic reticulum (ER) stress linked with impaired unfolded protein response (Figure 1). Also, DNA damage, mitochondrial impairment, changes in chromatin structure resulting mainly from its epigenetic modifications, and oncogene activation may contribute to senescence.

Senescent cells exhibit the senescence-associated secretory phenotype (SASP), marked by morphological changes and the direct release of various molecules, including cytokines, chemokines, growth factors, and proteases that contribute to chronic inflammation and tissue remodeling (Figure 1) (Wang et al., 2024). Senescent cells may also package signaling molecules into extracellular vesicles, such as exosomes and microvesicles, which can travel farther than soluble molecules and thereby influence more distant targets. Senescent cells also display metabolic perturbations, a persistent DNA damage response (DDR), changes in chromatin structure that inhibit gene expression (heterochromatin foci), mitochondrial impairment, and other features resulting from the action of senescence-inducing factors. Senescent cells amplify RONS signaling and are resistant to apoptosis (Deryabin et al., 2021).

Aging is a significant risk factor for cardiovascular disease (CVD). However, the term “aging” can refer to various concepts, including chronological, biological, oxidative, epigenetic, and social aging. The strong link between CVD and chronological aging has been clearly established. Moreover, a high burden of senescent cells correlates with CVD onset and progression (Kumar et al., 2024). However, chronological aging is associated with the accumulation of senescent cells due to replicative senescence and stress. Senescent cells release factors that contribute to tissue dysfunction and age-related disease, typical of advanced age. On the other hand, chronological aging increases stress and damage that induce senescence, thereby supporting the aging process. Removing senescent cells with senolytic drugs in experimental animals extended their health span (Robbins et al., 2021). Multiple recent reviews emphasize that cellular senescence is an independent risk factor for many cardiovascular diseases, including hypertension, atherosclerosis, MI, HF, and arrhythmias. Senescent cells accumulate with age and drive disease progression (Xu et al., 2025). Even though senescent cells represent only a small fraction of tissue cells, they exert a disproportionately large systemic impact through endocrine and paracrine effects (Wu et al., 2025).

As mentioned, senescent cells adopt SASP that drives chronic inflammation, fibrosis, remodeling of extracellular matrix (ECM), endothelial dysfunction and propagation of senescence to neighboring cells, making SASP a core mechanism linking senescence to cardiovascular dysfunction across various cell types, including cardiomyocytes, endothelial cells, fibroblasts, and vascular smooth muscle cells (Wu et al., 2025). The main molecular mechanisms linking senescence to CVD are oxidative stress and mitochondrial dysfunction, DNA damage, including telomere attrition, activation of the p53/p21 and p16INK4a signaling, metabolic impairment and epigenetic dysregulation (Chang et al., 2025; Ding et al., 2020; Grootaert, 2024; Hall and Lesniewski, 2024; Peoples et al., 2019). Senescent endothelial cells and vascular smooth muscle cells promote plaque formation, inflammation, and instability, contributing to atherosclerosis (Hall and Lesniewski, 2024). Accumulation of senescent cardiomyocytes and fibroblasts worsens post-MI remodeling and impairs regeneration (Redgrave et al., 2023). Senescence of vascular wall cells alters vascular tone, stiffness, and endothelial nitric oxide production (Afsar and Afsar, 2023). Arrhythmias, especially atrial fibrillation, have a strong and increasingly well-documented connection to cellular senescence. Recent research shows that senescent cardiac cells actively create a pro-arrhythmic environment through inflammation, calcium dysregulation, fibrosis, and electrical remodeling. The role of senescence in HF pathogenesis will be described in more detail in the subsequent section.

Senescent cell populations are heterogeneous, and this heterogeneity matters for senescence-related effects in CVD. Single-cell sequencing reveals distinct phenotypes with different functional roles, including pro-inflammatory and reparative (Mazan-Mamczarz et al., 2025). This variability affects treatment choices and disease progression. Therapeutic implications of senescence-related mechanisms in CVD pathogenesis are based on senolytics, which destroy senescent cells, and senomorphics, which suppress SASP and senescence signaling (Afsar and Afsar, 2023). Senolytics improve myocardial remodeling, enhance renal and vascular function, and reduce fibrosis and inflammation, while senomorphics reduce inflammation and modulate the MTOR, AMPK, and SIRT1 pathways. These approaches are considered to be compatible with regenerative methods, such as mesenchymal stem/stromal cell (MSC)- and induced pluripotent stem cell (iPSC)-based therapies (Chang et al., 2025).

In summary, cellular senescence is not just a passive byproduct of aging, but an active driver of cardiovascular disease development. Its effects are primarily mediated through SASP-induced inflammation, metabolic dysfunction, fibrosis, endothelial injury, and tissue remodeling.

## 4 Senescence in heart failure

Cellular senescence has emerged as a key mechanism driving the structural, metabolic, and inflammatory decline observed in chronic heart failure (CHF) (Zhao et al., 2025). Senescent cells accumulate in aging and stressed heart muscle, where they drive a SASP that releases pro-inflammatory cytokines, chemokines, and enzymes that modify the ECs, with strong local and systemic effects (Luan et al., 2024). This ongoing inflammation, supported by immunosenescence and continuous immune cell recruitment, worsens heart damage, causes endothelial dysfunction, and triggers secondary senescence in various cardiac cell types, including endothelial cells, fibroblasts, and cardiomyocytes (Młynarska et al., 2025). At the same time, SASP-driven activation of fibroblasts and abnormal ECM remodeling lead to increased ventricular stiffness and fibrosis, reducing heart compliance and worsening diastolic filling–hallmarks of heart-failure syndromes, especially HFpEF (Redgrave et al., 2025). Senescent cardiomyocytes further contribute to the decline in systolic function through mitochondrial damage, decreased contractility, and metabolic disruption, all of which reduce cardiac output and lead to fatigue and exercise intolerance in patients with CHF. Additionally, senescence-related endothelial cell inflammation and microvascular loss impair coronary blood flow and oxygen delivery, worsening symptoms such as shortness of breath, angina-like discomfort, and fluid retention by disrupting cardiac energy metabolism and elevating filling pressures (Zhang S. Y. et al., 2025). Clinical evidence shows an association between markers of cellular senescence and worse outcomes in ischemic and non-ischemic cardiomyopathies, including HFrEF and HFpEF (Evangelou et al., 2023). Senescent cells contribute to major HF phenotypes. In ischemic HF, senescent endothelial and smooth-muscle cells promote atherosclerosis, plaque instability, impaired angiogenesis, and worse myocardial recovery after infarction (Wu et al., 2021). In non-ischemic HF, senescence in cardiomyocytes and fibroblasts contributes to dilated and hypertrophic cardiomyopathies and maladaptive ECM remodeling, promoting fibrotic HF progression (Luan et al., 2024). Overall, these interconnected processes–chronic inflammation, fibrosis-related stiffness, declining contractility, and microvascular dysfunction–form a unified cascade that directly results in the main clinical signs of chronic heart failure, such as fatigue, shortness of breath, and swelling (Wu et al., 2025). In addition, senescence is implicated in anthracycline and radiotherapy-induced cardiotoxicity, contributing to long-term HF in oncology patients (Piegari et al., 2013; Zeng et al., 2022).

From a molecular standpoint, HF pathogenesis is increasingly viewed as arising from stress-triggered cellular aging in various cardiac and vascular cells. This begins with genotoxic and mitochondrial damage, telomere shortening, and oxidative stress, which activate DNA damage checkpoints (p53→p21 and p16INK4a→Rb), leading to cell resistance to apoptosis and pro-inflammatory responses (Chen et al., 2022). In heart muscle cells, senescence stems from mitochondrial dysfunction and defective mitophagy, leading to energy failure, weakened contraction, and harmful secretions (SASP) (Redgrave et al., 2023). After a heart attack, genetic or pharmacological inhibition of p16 improves heart remodeling and function, highlighting the role of senescent cells in HF growth. Chemotherapy and radiation injuries show upstream triggers and downstream effects: doxorubicin, for instance, causes persistent DNA damage and senescence in heart cells, and removing senescent cells restores heart function in mice, linking SASP inflammation to late cardiomyopathy and HF (Lérida-Viso et al., 2022; Linders et al., 2023). Thoracic radiation accelerates aging features in cardiomyocytes via mitochondrial bioenergetic defects linked to acetylation and induces endothelial senescence through DDR-NO signaling, leading to fibrosis, microvascular loss, and both diastolic and systolic HF symptoms (Nagane et al., 2021). In small blood vessels, senescent endothelial cells reduce NO availability, increase adhesion molecule expression, and release SASP factors that impair neovascularization and blood flow (Wan et al., 2024). Senescent vascular smooth muscle cells destabilize plaques and change artery walls, worsening ischemia, hindering repair, and promoting adverse heart remodeling and HF (Suda et al., 2023). In cardiac tissue, SASP cytokines (such as IL-6 and TGF-β) and enzymes from senescent cells and immune cells promote fibrosis and stiffening of ECM. Studies show that doxorubicin and stress pathways induce p16/p21-positive senescence; blocking these reduces senescence markers and improves heart function, linking cellular senescence mechanisms to fibrotic HF development (Huang et al., 2021).

**FIGURE 2:**
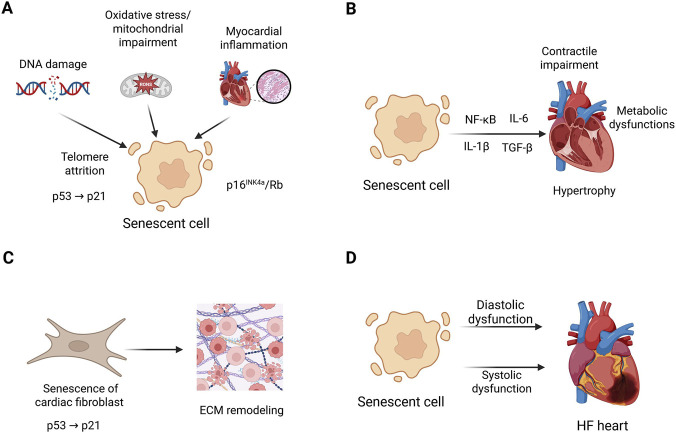
Structural and functional decline in senescence relevant heart failure (HF). Cellular senescence in cardiac and vascular cells is initiated by stresses such as DNA damage, oxidative stress/mitochondrial impairment, leading to overproduction of reactive oxygen and nitrogen species (RONS), and chronic inflammation. These stimuli activate signaling pathways, including p53/p21 and p16INK4a, leading to stable cell-cycle arrest **(A)**. Senescent cells are characterized by senescence-associated secretory phenotype (SASP) and release SASP mediators, including interleukins 6 and 1b (IL-6, IL-1β), transforming growth factor beta (TGF-β), proteases, that alter cardiomyocyte physiology by promoting hypertrophy, contractile impairment, metabolic dysfunction, and further propagation of senescence signals **(B)**. Senescent cardiac fibroblasts influence extracellular matrix (ECM) dynamics by shifting toward fibrosis, stiffening, and disorganized ECM deposition, contributing to impaired diastolic compliance and altered tissue architecture **(C)**. Accumulation of senescent cells and SASP-driven tissue changes contribute to both diastolic dysfunction, including HF with preserved ejection fraction through fibrosis and stiffness and systolic dysfunction, including HF with preserved ejection fraction through cardiomyocyte impairment and adverse remodeling **(D)**. Created in https://BioRender.com.

These findings reveal a complex, SASP-active network centered around DDR and mitochondrial stress, endothelial-microvascular failure, and fibroblast-ECM changes, providing insight into how senescence drives the structural and functional decline in HF (Figure 2).

## 5 Interplay between SIRT1 and senescence in heart failure

An increasing body of research identifies SIRT1 as a key molecular link between cellular senescence and the development of HF. SIRT1 levels decrease with age in both humans and animal models, thereby impairing endothelial function, heightening susceptibility to oxidative stress, and accelerating vascular aging, all of which contribute to HF (Ministrini et al., 2021). In the heart muscle, SIRT1 regulates mitochondrial homeostasis, including mitophagy, biogenesis, and metabolic efficiency; when SIRT1 levels are reduced, these processes are disrupted, leading to mitochondrial dysfunction, energy deficits, and increased oxidative stress, all of which are markers of cardiomyocyte aging involved in HF (Chen S et al., 2025). Senescent cardiomyocytes and endothelial cells also promote inflammation and fibrosis through the SASP, a process that worsens when SIRT1 levels decrease, further contributing to ventricular stiffening, microvascular rarefaction, and worsening contractile function (Chang et al., 2025; Camacho-Encina et al., 2024).

A key factor accelerating this decline is that senescence actively promotes the breakdown of SIRT1 protein. Under stress-induced premature senescence (SIPS), SIRT1 becomes a target for the cell-cycle–regulated ubiquitin ligase complex (APC/C-Cdh1)–mediated ubiquitination, leading to its proteasomal degradation; however, active regulator of SIRT1 (AROS) prevents this loss by competitively stabilizing SIRT1 (Lee et al., 2023). This APC/C-Cdh1-AROS axis controls SIRT1 levels and the upstream regulation of SASP genes, including interleukins IL-1 and IL-6 genes, making SIRT1 degradation a crucial amplifier of senescence-related inflammation that impacts structures involved in HF (Lee et al., 2023).

Cardiac stressors, such as ischemia-reperfusion (I/R), further demonstrate the effects of SIRT1 protein instability (Gu et al., 2014). Recent mechanistic research shows that checkpoint kinase 1 (CHK1) phosphorylates SIRT1 at Thr530, preventing SMAD-specific E3 ubiquitin protein ligase 2 (SMURF2)-mediated ubiquitination, thereby maintaining SIRT1 levels, mitochondrial biogenesis, and mitophagy. Loss of CHK1 or impaired phosphorylation increases SIRT1 degradation, leading to mitochondrial dysfunction and worsening I/R-induced cardiac injury. This underscores that SIRT1 degradation directly accelerates myocardial damage in HF (Yang TT. et al., 2025).

Chemotherapy-induced cardiomyopathy provides another well-defined example of senescence-related SIRT1 depletion. Doxorubicin, a powerful inducer of DNA damage and senescence, decreases SIRT1 levels in cardiomyocytes, leading to mitochondrial dysfunction, reduced DNA repair ability, and caspase activation (Kuno et al., 2013; Kuno et al., 2023). Doxorubicin also increases miR-34a, a senescence-associated microRNA that directly represses SIRT1, activates p66Shc signaling, a key regulator of oxidative stress responses, autophagy, apoptosis, and aging (Powell et al., 2025). This promotes apoptosis and senescence in cardiomyocytes and cardiac progenitor cells; blocking miR-34a restores SIRT1, reduces p53 acetylation and p16INK4a expression, and enhances cardiac cell survival (Piegari et al., 2016; Zhu et al., 2017).

Taken together, these data support a model in which SIRT1 functions as a molecular brake on cardiovascular aging and HF development by limiting senescence, preserving mitochondrial efficiency, suppressing SASP-mediated inflammation, and maintaining endothelial health. When SIRT1 declines, these protective effects are lost, allowing senescence to drive harmful remodeling, inflammation, microvascular dysfunction, and energy failure that lead to symptomatic HF. Therapeutically, using SIRT1-activating compounds and NAD^+^-restoring treatments to improve mitochondrial function and decrease senescence-related traits highlights SIRT1 as a promising target for slowing or reversing HF progression (Chang et al., 2025; Chen S et al., 2025). The next section examines this issue in more detail.

## 6 Perspectives on the therapeutic exploitation of SIRT1 modulators in senescence-related heart failure

Senescence-related HF is caused by mitochondrial dysfunction, impaired autophagy, chronic inflammation, metabolic inflexibility, and maladaptive hypertrophy. SIRT1 directly influences each of these processes, and many modern HF treatments, whether or not they are designed with aging biology in mind, target pathways that overlap with or activate SIRT1 (Zhou et al., 2024).

Sodium-glucose cotransporter-2 (SGLT2) is a kidney transport protein responsible for reabsorbing most of the glucose filtered by the kidneys (Sano et al., 2020). Large clinical studies showed that SGLT2 inhibitors improved heart outcomes, also in HF, even in people without diabetes (Ibrahim et al., 2025). SGLT2 inhibitors, including empagliflozin and dapagliflozin, trigger a fasting mimicry metabolic state that activates SIRT1, PGC-1α, and fibroblast growth factor 21 (FGF21), promoting autophagy and reducing oxidative stress (Packer, 2020). This same SIRT1/PGC-1α axis is strongly beneficial in senescence-related HF, where mitochondrial dysfunction and impaired organelle turnover dominate (Zhang X. et al., 2025).

Therapeutic strategies in HF are increasingly focused on correcting mitochondrial dysfunctions (Parker et al., 2025). SIRT1 enhances mitochondrial biogenesis and energy balance through its effects on PGC-1α, PPARα, estrogen-related nuclear receptor alpha (ERRα), and other metabolic regulators, aligning with the objectives of metabolic HF therapies (Zhou et al., 2024). Therefore, current metabolic therapies indirectly utilize SIRT1-like mechanisms, even if SIRT1 itself is not directly targeted.

As mentioned, autophagy failure is a hallmark of cardiac aging and senescence. Activation of SIRT1/PGC-1α/AMPK improves autophagic flux, removing dysfunctional mitochondria and misfolded proteins–a key therapeutic target for future HF treatment (Liang et al., 2020). Because autophagy gene silencing eliminates longevity benefits and worsens cardiomyopathy, agents that activate SIRT1-dependent autophagy are strong candidates for treating senescence-related heart failure.

SIRT1 reduces inflammation by deacetylating transcription factors, including FOXO1, NFκB-related regulators, hypoxia-inducible factor 1 alpha subunit (HIF-1α), and p53, which control oxidative stress and inflammatory cytokines (SIRT1, 2026). Current HF therapies, such as angiotensin-converting enzyme (ACE) inhibitors/angiotensin II receptor blockers (ARBs) (renin–angiotensin–aldosterone system (RAAS) inhibition, angiotensin receptor–neprilysin inhibitor (ARNI, sacubitril/valsartan), and beta blockers, all indirectly reduce neurohormonal and oxidative stress (Álvarez-Zaballos and Martínez-Sellés, 2023; Lien, 2025). SIRT1 activation would work together with these therapies by further reducing oxidative stress, enhancing mitochondrial redox balance, and decreasing SASP-like inflammatory signaling in senescent myocardium. Therefore, SIRT1-based interventions could support standard neurohormonal blockade. SIRT1 activation inhibits maladaptive hypertrophic signaling by blocking the AKT/MTORC1 pathway and promotes cellular survival mechanisms over growth (Shuai et al., 2024). This aligns with the effects of ARNI, which reduces fibrosis and remodeling, and mineralocorticoid receptor antagonists (MRAs), which decrease hypertrophy and inflammation (Abbou et al., 2021; Chen X. et al., 2025). Future SIRT1-modulating therapies may strengthen these effects by directly targeting the longevity gene axis that regulates cardiomyocyte survival versus hypertrophy.

Although not yet a standard in clinical practice, senescence-targeting therapies (senolytics, SASP inhibitors, NAD^+^ boosters) are a rapidly developing class. SIRT1 activation acts as an inherent senomorphic mechanism, reducing SASP, enhancing mtQC, and delaying cardiac aging (Zhang S. Y. et al., 2025). Therefore, SIRT1 modulators naturally fit into the next-generation therapeutic landscape targeting aging biology, which is becoming increasingly important in HF as populations grow older.

Since SIRT1 is NAD^+^-dependent, age-related NAD^+^ decline decreases SIRT1 activity, which in turn reduces its cardioprotective effects. Therefore, restoring NAD^+^ levels may restore SIRT1’s cardioprotective functions. This aligns with improvements in mitochondrial health, reduced cardiac aging, and increased autophagy (Zhang S. Y. et al., 2025). These approaches could be promising for the therapy of senescence-related HF (Table 1).

**TABLE 1:** Potential of sirtuin 1 in senescence-related heart failure therapy.

HF^a^-related pathology	Current therapy	Role of SIRT1
Mitochondrial dysfunction	SGLT2 inhibitors, metabolic modulators	SIRT1 restores mtQC
Oxidative stress and inflammation	ACEi/ARB/ARNI, beta blockers	SIRT1 deacetylates redox and inflammatory regulators, lowering oxidative burden
Impaired autophagy	Indirect benefits from SGLT2i, caloric restriction mimetics	SIRT1/PGC-1α activation enhances autophagy, clearing dysfunctional organelles
Senescence-driven remodeling	MRAs, ARNI	SIRT1 suppresses MTORC1 and maladaptive hypertrophy, promoting cardiomyocyte survival

^a^ Abbreviations: ACEi, angiotensin-converting enzyme inhibitors; ARBs, angiotensin II receptor blockers; ARNI, angiotensin receptor–neprilysin inhibitor; HF, heart failure; MRAs, mineralocorticoid receptor antagonists; MTORC1, mechanistic target of rapamycin complex-1; PGC-1α, peroxisome proliferator-activated receptor gamma coactivator 1-alpha; SGLT2, sodium-glucose cotransporter-2; SIRT1, sirtuin 1

## 7 Conclusion, perspectives, and outstanding questions

Converging evidence points to SIRT1 as a key regulator of cardiovascular aging: it protects endothelial function, inhibits senescence programs, and sustains mitochondrial health in cardiac cells. Aging and cardiometabolic stress decrease SIRT1 levels and activity, which promotes senescence along with SASP, mitochondrial ROS, and DNA-damage signaling. These changes accelerate adverse remodeling and HF progression. Understanding where, when, and how SIRT1 loss connects with senescence across cell types, including cardiomyocytes, endothelium, fibroblasts, and immune cells, is a practical Frontier for HF biology and therapeutic development.

Single-cell and spatial multi-omics should map SIRT1 expression and activity, along with NAD^+^ levels, and senescence markers (p16/p21, SASP modules) across cardiomyocytes, endothelial cells, fibroblasts, and immune cells, from pre-HF to advanced disease. Prior reviews already highlight SIRT1 loss associated with endothelial dysfunction and senescence in humans and models, emphasizing mitochondrial and senescence programs as central to cardiac aging; the next step is to clarify the sequence of events and cellular cross-talk in human HF tissues (Zeng et al., 2020).

Further studies are needed on dissecting post-translational SIRT1 degradation as a senescence amplifier in the failing heart. It is also important to take a closer look at the significance of SIRT1 loss for organelle-stress circuits mitochondria-lysosome-autophagy and paracrine spread of senescence.

From a clinical perspective, interventions that restore NAD^+^ or imitate nutrient deprivation, such as SGLT2 inhibitors that activate the SIRT1/PGC-1α/FGF21 pathway, warrant rigorous mechanistic evaluation of senescence, beyond just clinical outcomes. Preclinical studies indicate that SIRT1 activation (for example, by resveratrol) protects the DNA damage response (H2AX deacetylation) and enhances LV function after cardiotoxic stress. However, sustained benefits in HF may require dual strategies: (1) stabilizing SIRT1 protein (such as increasing CHK1 phosphorylation or preventing cadherin 1 (Cdh1)/SMURF2-mediated ubiquitination), and (2) boosting SIRT1 enzymatic activity or replenishing NAD^+^ (Gu et al., 2014; Sin et al., 2014). Combining SIRT1-centric strategies with senolytics to reduce senescent cell burden or senomorphics to suppress SASP could lead to additional improvements in diastolic stiffness, microvascular function, and energetics.

SIRT1 sits at the intersection of mitochondrial health, inflammation control, metabolic regulation, autophagy, and anti-aging processes, all of which are closely linked to senescence-related HF. Therefore, existing HF treatments already target SIRT1-related pathways, such as SGLT2 inhibitors and anti-remodeling agents. SIRT1 modulators could improve the effectiveness of current standard therapies. Future HF treatments are expected to increasingly harness longevity gene signaling, positioning SIRT1 as a key focus of next-generation HF therapeutics.

The SIRT1-senescence axis is no longer peripheral; it is now a central driver of HF pathogenesis, linking DNA damage, mitochondrial failure, endothelial dysfunction, fibrosis, and inflammatory remodeling. Future research that stabilizes and reactivates SIRT1 while halting senescence spread offers a clear, mechanism-based approach to changing HF outcomes and opens new opportunities in HF treatment.

The future studies should address the following outstanding questions important for both basic research and therapeutic perspectives: (1) does SIRT1 decline precede senescence (causal), or does senescence-induced SASP suppress SIRT1 secondarily? (2) which myocardial compartments (microvasculature vs. myocyte vs. interstitium) initiate this cascade in HFpEF versus HFrEF? (3) What molecular signals trigger the switch from SIRT1 downregulation to full senescence commitment in cardiac cells? (4) how do post-translational SIRT1 degradation pathways integrate under chronic HF stress? (5) what determines cardiomyocyte versus endothelial susceptibility to SIRT1-regulated senescence? (6) how does the SIRT1-miR-34a axis operate in human HF beyond cardiotoxicity models? (7) does SIRT1-regulated lysosomal dysfunction occur in native HF and not just in drug cardiotoxicity? (8) how do NAD^+^ depletion and metabolic dysregulation shape SIRT1-senescence feedback loops in HF? (9) what combination therapies best exploit the SIRT1-senescence axis?

We aimed to identify current knowledge gaps regarding the role of SIRT1 in senescence-mediated pathogenesis. Senescent cell populations in HF include cardiomyocytes, cardiac fibroblasts, endothelial cells, and vascular smooth muscle cells. Each cell type employs distinct SIRT1-dependent signaling networks. However, systematic mapping of SIRT1 actions across different cardiac cell types is lacking. While SIRT1 generally appears to be cardioprotective, in some contexts, SIRT1-mediated pathways may promote cell survival at the expense of allowing dysfunctional or pre-senescent cells to persist. No studies have determined whether SIRT1 activation might worsen remodeling in late-stage HF by supporting the survival of senescent cardiomyocytes. Another gap is the limited clinical translation and biomarker development, as most evidence derives from rodent or cellular models. Consequently, there is no reliable biomarker of cardiac SIRT1 activity in humans, a limited understanding of NAD^+^ metabolism in the aged human myocardium, and poor pharmacokinetics of current SIRT1 activators, as noted in recent reviews. Moreover, SIRT1 regulates circadian rhythm, inflammation, and metabolic homeostasis, all of which intersect with senescence. Yet these axes remain largely unstudied in HF models, especially regarding how circadian regulation influences cardiac senescence (Molnár et al., 2023; Stojanović et al., 2025).
